# Notable concerns in methodology and conclusions of the Wang et al. Meta-analysis in *BMJ* by the American Academy of Pain Medicine

**DOI:** 10.1093/pm/pnaf036

**Published:** 2025-03-28

**Authors:** Nathaniel M Schuster, Mustafa Broachwala, Farshad M Ahadian, Charles E Argoff, Steven P Cohen, Shravani Durbhakula, Amitabh Gulati, Robert W Hurley, Lynn Kohan, Zachary L McCormick, Sayed E Wahezi, Antje M Barreveld

**Affiliations:** Center for Pain Medicine, Department of Anesthesiology, University of California, San Diego, San Diego, CA, United States; Center for Pain Medicine, Department of Anesthesiology, University of California, San Diego, San Diego, CA, United States; Center for Pain Medicine, Department of Anesthesiology, University of California, San Diego, San Diego, CA, United States; Department of Neurology, Albany Medical College, Albany, NY, United States; Departments of Anesthesiology, Neurology, Physical Medicine & Rehabilitation, Psychiatry, and Neurological Surgery, Northwestern University Feinberg School of Medicine, Chicago, IL, United States; Walter Reed National Military Medical Center, Uniformed Services University of the Health Sciences, Bethesda, MD, United States; Department of Anesthesiology, Vanderbilt University School of Medicine, Nashville, TN, United States; Department of Anesthesiology, Memorial Sloan Kettering Cancer Center, New York, NY, United States; Pain Outcomes Lab, Pain Service Line—Atrium Health Wake Forest Baptist, Departments of Anesthesiology, Translational Neuroscience, and Public Health Sciences, Wake Forest University School, Winston-Salem, NC, United States; Department of Anesthesiology, University of Virginia Health System, Charlottesville, VA, United States; Department of Physical Medicine and Rehabilitation, University of Utah School of Medicine, Salt Lake City, UT, United States; Department of Pain Medicine, Montefiore Multidisciplinary Pain Program, Bronx, NY, United States; Tufts University School of Medicine, Newton-Wellesley Hospital, Newton, MA, United States

As pain medicine physicians, interventionalists, researchers, leaders, and members of the American Academy of Pain Medicine, we have significant methodological concerns regarding the erroneous conclusions raised in the *BMJ* article entitled “Common interventional procedures for chronic non-cancer spine pain: a systematic review and network meta-analysis of randomized trials” and its accompanying guidelines and editorial.^1^ The *BMJ* article assessed a heterogenous array of interventional spine procedures (some commonly performed while others are not) and arrived at overgeneralized and faulty conclusions. As pain medicine experts who routinely witness the often life-changing impact of interventional procedures, we caution against the *BMJ* article’s flawed conclusions that risk restricting patient access to treatments that allow many of our patients to continue working, caring for their families, and living active lives.

Given the unrealistic aim of the analysis to evaluate a broad and heterogeneous number of interventional procedures and a vast amount of literature, much of the substance of the work was relegated to the supplements. Review of the supplements reveal more limitations than can be covered in one editorial. We will outline a few of these limitations here:

1. *Relevant positive studies were not included in generating their conclusions*. For instance, the authors’ forest plots did not include the seminal Lord *et al.* randomized, sham-controlled trial demonstrating the efficacy of cervical facet radiofrequency ablation that was published in the *New England Journal of Medicine*.^2^ While the authors were unable to combine data from the Lord *et al.* study with other similar studies, this reflects a limitation of a meta-analysis, not treatment inefficacy.
2. *Differing and evolving interventional techniques were inappropriately grouped together*. The varying results of lumbar medial branch thermal radiofrequency ablation randomized controlled trials (RCTs) are known to differ based on the rigor of their patient selection and technique.^3^ Lumbar facet and sacroiliac complex radiofrequency ablation techniques have evolved over time. Wang *et al.*’s meta-analysis included lumbar radiofrequency ablation studies that used small-gauge electrodes placed perpendicular to the target nerves, techniques which are now known to be insufficient to produce a favorable treatment outcome, as well as studies that used less rigorous patient selection, which has been demonstrated to result in poorer outcomes in clinical trials.^3^ Wang *et al.*’s meta-analysis also included sacroiliac complex radiofrequency ablation techniques that used needle placements now known not to be anatomically valid.^4^ This meta-analysis inappropriately grouped radiofrequency ablations at 80°C with mechanistically different, non-ablative pulsed radiofrequency treatments at 42°C, which are known to be less effective and provided shorter duration of benefit. Grouping studies with inappropriate patient selection and different procedural techniques dilutes the treatment effect observed in studies with optimal methodology.
3. *Relevant, contemporary and effective spine interventional procedures were not included in this overarching analysis*. Although the titles of the Wang *et al.* systematic review and meta-analysis and Busse *et al.* guidelines both begin with the word “Common,” the articles did not include more commonly performed procedures with robust evidence of effectiveness, while including irrelevant, ineffective, and uncommon procedures. The analyses did not include contemporary effective interventional procedures with robust evidence such as basivertebral nerve ablation, which is supported by a positive double-blind, sham-controlled RCT with long-term benefit at 5 years, a comparative-effectiveness RCT comparing the treatment to standard-of-care, and positive healthcare utilization and cost-effectiveness data.^5–8^ Additionally, the authors do not discuss percutaneous interlaminar ligamentum flavum decompression, with positive RCTs including a comparative effectiveness RCT demonstrating its superiority to epidural steroid injections with long-term benefit at 2 years and another RCT demonstrating its superiority to conventional medical management.^9^^,^^10^
4. *Irrelevant and ineffective spine interventional procedures were included in this analysis*. Meanwhile, the authors included in their analysis treatments that never progressed to common use in clinical practice, such as intra-articular atlanto-occipital joint pulsed radiofrequency, intra-articular facet and sacroiliac pulsed radiofrequency, and dorsal root ganglion non-ablative radiofrequency treatments.
5. *Data were incorrectly abstracted*. For example, the authors incorrectly abstracted that the atlanto-occipital joint pulsed radiofrequency study was studying a facet procedure for non-specific axial neck pain.

It is evident that the meta-analysis and guidelines did not have adequate involvement from spine interventional experts to identify which procedures are “common” and to identify critical differences in procedural techniques and patient selection.

*Additionally, the authors claim that these treatment should only be offered in RCTs—which would result in many patients with conditions treatable by interventions being treated surgically or with opioids, being undertreated, or not being treated at all*. Not all patients would be interested in participating in clinical trials or able to access clinical trial sites. Of patients who are interested in participating in any given clinical trial, typically <20% of screened patients qualify for and are enrolled.

Finally, the authors neglected to consider unintended consequences. There are many patients who experience meaningful relief with interventional procedures after numerous other modalities have not improved those patients’ pain. We have seen patients who have been harmed by curtailed access to interventional procedures. When payers restrict coverage of interventional procedures, those with the financial means pay for access to interventional treatments, while those less fortunate suffer. When patients do not have access to interventional pain procedures, some patients resort to treatment with opioids (whether prescription or otherwise) and some proceed to surgeries that could otherwise be avoided, at even greater costs to society.

Though we recognize the *BMJ*’s intentions with “Rapid Recommendations” to produce clinical practice guidelines accompanying meta-analyses, clinical guidelines based on a severely flawed network meta-analysis, and with inadequate contributions from experts knowledgeable in which procedures are common and the differences in techniques used in published studies, makes this guideline fraught for misinterpretation and misapplication. While we commend their efforts to include four patients in the development of the guidelines, these publications would have been well-served by similarly including four interventional pain experts in both the network meta-analysis design and implementation and guidelines development.

The American Academy of Pain Medicine believes that pain care should be multidisciplinary and patient-centric. Interventional spine procedures can provide life-changing benefits in appropriately selected patients and allow many patients to remain in the workforce and live their best lives. Pain care must be individualized to each patient, balanced with available experts and access, and patient preferences and values. Pain medicine requires a nuanced, individualized approach that cannot be adequately captured by a flawed, overgeneralized meta-analysis and guideline.

The high impact and cost of chronic pain for individuals and society justifies the need for increased funding for robust pain research. Interventional procedures are a critical part of patient-centric pain care, and with innovation and expanded research, interventional procedures will continue to evolve to further improve lives and decrease disability.
